# Harvesting the free fibular graft: A modified approach

**DOI:** 10.4103/0019-5413.73657

**Published:** 2011

**Authors:** Amitava Narayan Mukherjee, Ananda Kisor Pal, Debashis Singharoy, Debadyuti Baksi, Chinmoy Nath

**Affiliations:** Department of Orthopedics, J.N. Roy General Hospital, Burdwan, India; 1 Department of Orthopedics, Burdwan Medical College, Burdwan, India; 2 Department of Orthopedics, K.P.C Medical College and Hospital, Jadavpur, India; 3 Department of Orthopedics, Calcutta Medical College and Hospital, Kolkata, India

**Keywords:** Fibular grafting, free fibular graft, minimal invasive fibular graft

## Abstract

**Background:**

The conventional technique of free non-vascularized fibular grafting is attended with some amount of morbidity and a long scar. We report a technique with little interference to the surrounding soft tissues to harvest more than one-third of whole length fibula.

**Patients and Methods:**

Thirty four patients of average age 23.5 years (range 8 to 51 years) having various pathologies like simple bone cysts (n=9), fibrous dysplasias (n=6), giant cell tumors (n=7), fracture non-union (n=10) and aneurysmal bone cysts (n=2) were taken up for the study. The fibula were harvested by two separate incisions, 1 cm each at proximal and distal extent of proposed donor site for taking out of graft after elevating the periosteum circumferentially using a periosteum stripper. Compression bandage and above knee plaster immobilization was applied to reduce the dead space collection.

**Results:**

The mean followup is 34 months. The patients were evaluated clinicoradiology. Thirty three patients showed good results. One patient had fair result due to delayed wound healing from hematoma which was treated surgically.

**Conclusion:**

The approach of harvesting fibula suggested by author reduces donor site morbidity and is safer than conventional approach.

## INTRODUCTION

Fibular grafting is a common procedure in orthopedic practice. The conventional technique of harvesting fibular graft by long incision results in increased morbidity and a long scar,^1^–^3^ weakness of extensor hallucis longus and ankle instability particularly when the required graft is long and extended into distal third.^4^–^6^ This fact gave rise to thought whether the same graft can be harvested in such a way, which can reduce the donor site morbidity especially where relatively longer (more than one-third of whole length) fibula is required. Hence, a biological approach for harvesting long free non-vascularized fibular graft with least interference of surrounding soft tissue maintaining periosteal sleeve (hence biological) by minimal invasive technique was attempted and critically evaluated in respect to recovery of satisfactory functions and development of complications compared to that of conventional technique.

## PATIENTS AND METHODS

We included the patients where longer fibular graft (more than 1/3 of whole length fibula) was required for filling the lesion. Thirty four patients of various bony lesions including, simple bone cysts (n=9), fibrous dysplasia (n=6), giant cell tumor (n=7), fracture non-union (n=10) and aneurysmal bone cysts (n=2) were included in study. The age of the patients varied from 8 to 51 years (average 23.5 years). We had four children having simple bone cysts in proximal humerus (three) and proximal femur (one). For tibial lesions, fibula of contralateral side was selected. The patients were excluded where either fibula or the surrounding soft tissues were unhealthy from injury or any pathological condition.

**Figure 1:**
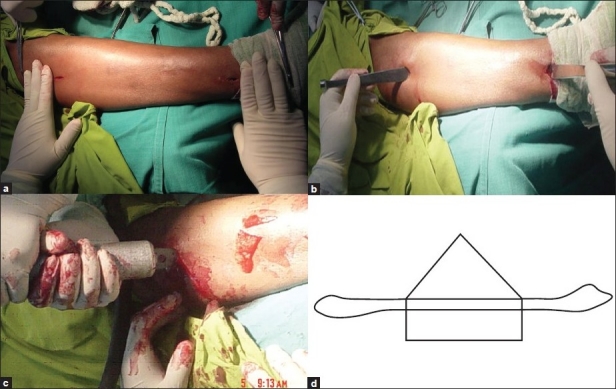
Per-operative photograph of minimal invasive operation technique of harvesting nonvascularized free fibular graft showing (a) the small proximal and distal incisions (b) elevation of periosteum from the proximal and the distal wounds with ordinary periosteum elevator as far as possible (c) roof head cut of proximal end of the graft and (d) schematic diagram of roof head cut

Preoperative assessment was done for the length of the graft required. Under tourniquet control, a sand bag was kept beneath the pelvis and a pillow beneath the leg. The proposed length of fibula was marked over the outer aspect of leg. Two incisions (lateral or anterolateral) were made, 1 cm each over the proximal and distal end of proposed length of the graft [Figure 1a]. Incision was made along the posterior margin of the fibula. The superficial and deep fasciae were divided. A plane between the soleus muscles posteriorly and peroneal muscles anteriorly, was developed. Conventional periosteum elevators were used to elevate the periosteum through the small incisions towards the midshaft of fibula as far as possible except the interosseous border. [Figure 1b]. Special care was taken for slow and steady use of periosteum elevator during elevation of the periosteum from the posterior surfaces of the midshaft because of nutrient vessels. After elevation of the periosteum maximally from the anterior (extensor), lateral (peroneal) and posterior (flexor) surfaces, the proximal and distal end of proposed fibula is cut as per roof head cut fashion [Figures 1c and d], which means length of the outer cortex is more than the length of the inner cortex.

**Figure 2:**
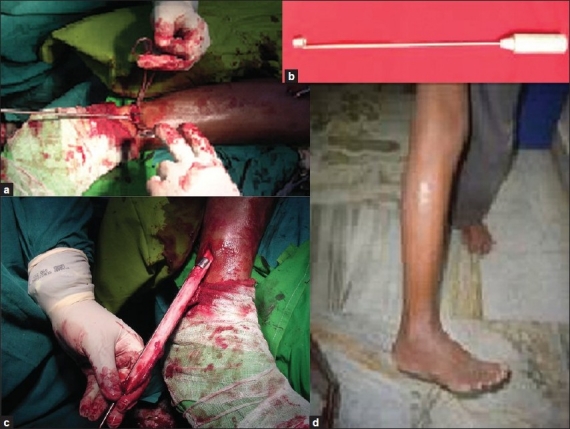
Per-operative photograph of operation technique of elevating rest of the periosteum from the cut fibular graft with special periosteum stripper (a), a special periosteum stripper (b), removal of whole fibular graft (c) and post operative clinical photograph showing highly cosmetic scar (d)

Then the distal cut end of the fibula is held by a bone holding forceps and gently pulled outside to get access of the interosseous border [Figure 2a]. Then the special periosteal stripper [Figure 2b] is introduced through the distal cut end keeping the anterior slit laterally and shaft side containing the circular periosteal stripper medially to protect the neurovascular structure. In this way periosteum is elevated all around from the fibula especially medial part of periosteum blended with interosseous membrane by upward advancement of the periosteal stripper [Figure 2a] with gentle 20 to 30° rotation and also avoid traction injury to neurovascular structures running proximal to distal ward especially the segmental branches of peroneal vessel. The graft was gently rotated inside to ensure that it was free from the surrounding soft tissues and then it was delivered preferably through the distal incision [Figure 2c] to avoid injury to neurovascular structures. Compression bandage was applied with above knee plaster slab till removal of the stitches maximally up to two weeks followed by active range of motion exercises of knee and ankle. Gradual weight bearing was allowed after three to four weeks postoperatively depending on local wound and main pathology.

## RESULTS

Patients were followed up for mean period of 34 months periodically every six weeks for first six months and then at three monthly interval. As there are no standard criteria for assessing donor site morbidity suitable for patients, we evolved our own criteria and used that for assessment of our patients clinically on the basis of pain at donor site, adjacent joint (knee and ankle) motions, wound healing and development of complications [Table 1].

**Table 1:** Criteria for assessment of results

Grade of result	Pain at donor site	Adjacent joint motions	Wound healing	Complications
Good	Nil	Full	Primary	Nil
Fair	Occasional	Partial restriction	Secondary	Present, managed with treatment
Poor	Constant	Significant stiffness or instability	Secondary with persistent sinus	Refractory complications

A questionnaire was given to all patients comprising simple questions like ability to squat, sitting crossed legged, getting up and down through stairs, enter inside the vehicle, carry heavy weight etc and asked to qualify whether they were able to perform those activities with difficulty or satisfactorily. Thirty three patients showed good results without pain, satisfactory recovery of adjacent joint (knee and ankle) motions without neuromuscular complications. There was no patients with knee stiffness or ankle instability. One patient had fair result due to development of hematoma, which were treated with drainage by removal of one stitch. Three out of four children showed regeneration of periosteum completely. All patients having minimal scar, had highly satisfactory results on cosmetic ground [Figure 2d].

## DISCUSSION

In this study, we used two small incisions for cutting the upper and lower end of proposed length of fibula. Here, utmost care was taken to avoid the damage of large and constant branches of peroneal vessels. As there are two constant major branches of peroneal vessel supplying the soleus running at a distance of 6-12 cm from fibular head, so the proximal incision was made 6-12 cm away from fibular head decreasing the chance of inadvertent injury to the same. Otherwise, if any vessel inadvertently came in the way was ligated.

Conventional periosteal elevator was used to elevate the periosteum from the anterior (extensor), lateral (peroneal) and posterior (flexor) surfaces except its medial part blended with interosseous membrane. The proposed periosteal stripper [Figure 2b] is indigenously designed by the author, manufactured by Calcutta Metallic Company Private Limited, Kolkata and applied for the patent. It is available in four sizes with diameter varied from 8, 9, 10 to 11 mm with flexible shaft having lengths varied from 260, 280, 300 to 320 mm respectively, based on the radiological and cadaveric study of fibula. It has a longitudinal slit of 2 mm between two flanges, which embrace the fibula. As the stripper remains adjacent to the bone during its advancement upward with gentle 20 to 30° rotation; all the neurovascular structures remain protected with global elevation of the periosteum from the harvesting fibula.

The ‘roof head cut’ of proximal and distal end of fibular graft ensured in easy and safe introduction of newly designed periosteal stripper and allowed less periosteal stripping of the medial aspect, thereby preserving the periosteum on interosseous border and facilitated easy delivery of graft preferably through distal incision. Roof head cut also left more length of fibula attached with interosseous membrane, which is especially advantageous distally for ankle stability. By this minimal invasive technique, injury of three to four fascio cutaneous branches of peroneal artery, which gives segmental musculoperiosteal vessel to the midshaft of fibula and also supplying the overlying skin and subcutaneous tissue was avoided. Thereby we had less hematoma, infection and wound dehiscence in our series. Overall, this minimally invasive technique causes minimal soft tissue handling with minimal complications and cosmetically superior compared to 10 to 24% donor site complications^6^–^10^ seen with conventional technique using a long incision. We have satisfactory regeneration of periosteum in three (75%) children similar to other workers experience.^5^

It did not cause weakness of any muscle (either Extensor Hallucis Longus or Peronei) as it did not require reconstruction of muscle compartment as in open conventional technique. There was no ankle instability as we avoided distal 8 to 10 cm fibula, which is the minimal length of distal fibula required for ankle stability.^6^ Our patients did not have any complaints regarding body weight transmission on the affected limb as seen by other workers.^7^ By this technique, we could harvest maximum 20 cm length of fibula in an adult whose total length of fibula was 36 cm and minimum of 10 cm fibula in a child whose total length of fibula was 25 cm having minimal donor site morbidity with high satisfaction. Thus, this biological method appears to be an attractive proposition for harvesting of relatively longer fibular graft in different pathological conditions.
